# Incidence of HPV and HPV related dysplasia in elderly women in Sweden

**DOI:** 10.1371/journal.pone.0229758

**Published:** 2020-03-20

**Authors:** Lars Lannér, Annika Kristina Lindström

**Affiliations:** 1 Department of Women’s and Children’s Health, Uppsala University, Uppsala, Sweden; 2 Clinical Research Center, Faculty of Medicine and Health, Örebro University, Örebro, Sweden; Rudjer Boskovic Institute, CROATIA

## Abstract

**Background:**

About one-third of the cervical cancer cases in Sweden occur in women over the age of 60. The primary aim of this study was to analyze the incidence of HPV, and HPV related dysplasia, in elderly women who had an HPV negative test at the age of 60 years or older.

**Methods:**

From October 2004 to June 2019, 1784 women aged 60–90 years were sampled for an HPV test when attending an outpatient gynecology clinic. Of these women, 827 HPV-negative women had two or more HPV tests at intervals of three months to eleven years (mean 3.2 years). The women with positive results had a repeat HPV test and cytology after 2.5 months on average. Those with a positive repeat HPV test were examined by colposcopy and biopsy.

**Findings:**

The overall prevalence of HPV was 5.4%, (95%CI 4.4–6.6, 96/1784). The incidence of HPV in the 827 women, who were HPV negative in their first test, was 2.4% (95%CI 1.5–3.8, n = 20). At the repeat test 1.2% remained positive (95%CI 0.6–2.3, n = 10). HPV-related dysplasia diagnosed by histology was found in 1.2% (95%CI 0.6–2.3, n = 10) of the 827 women. CIN2+ was found in 0.5% (95%CI 0.2–1.3, n = 4). In the repeat HPV test 52.6% 10/19) were HPV positive. The time between an HPV negative test and an HPV positive test and CIN2+ was on average 45.5 months (range 10–85 months). The positive predictive value (PPV) for CIN2+ was 20.0% in the first positive HPV test and 40.0% in the repeat HPV test. The women with CIN2+ had normal cytology. No cancer or glandular dysplasia was detected.

**Interpretation:**

In this study older HPV-negative women were at risk of becoming HPV positive. Among the women who were HPV positive in a repeat test, there was a high risk of dysplasia.

## Introduction

The incidence of cervical cancer (CC) in Sweden has increased over the past ten years despite a screening program with a coverage of more than 80% for the ages screened [1]. In Sweden, about 30% of CC cases occur in women over 60 and the mortality rate is about 70% in this age group [2, 3]. Cervical cancer in women above the age of 65 is usually discovered at advanced stages and the prognosis is poor [4]. Women previously treated for cervical intraepithelial neoplasia grade 3 (CIN 3), are at increased risk of developing and dying from cervical or vaginal cancer, and the risk accelerates above 60 years of age [5]. A combination of organized and opportunistic Pap smear screening has reduced the incidence of squamous cell cancer by around 70%, in cohorts most regularly screened [6]. Older women are not included in screening programs for cervical cancer. The 2015 Swedish cervical screening guidelines are under implementation [7]. Since 2017, HPV based screening for women of 30–64 years of age is recommended, but most parts of Sweden still have screening only until the age of 60 [7]. The part of Sweden where this study was performed introduced primary HPV screening up to the age of 64 in 2018. Over the past century, the average life expectancy for Swedish women has increased from 55 to 84 years [8]. Many women over 65 are healthy, continue to work, and have an active sex life [9].

In post-menopausal women, due to hormonal changes, the transformation zone where precursor lesions develop is situated in the cervical canal and is therefore not accessible for proper examination and sampling [10, 11]. As a consequence, Pap smear for conventional cytology or liquid-based cytology (LBC), has a low sensitivity and diagnostic surveillance with colposcopy for a biopsy is of little value [12, 13]. Examination with biopsies and cervical curettage for histology has a higher sensitivity for the detection of dysplasia in post-menopausal women and should be the method of choice [11, 13].

There is no consensus as to what age screening for cervical cancer should stop. According to European Guidelines, screening with cytology or HPV could be discontinued around 60–65 years, given that the woman has had a recent negative test (supporting level of evidence: expert opinion) [14]. To our knowledge, there is limited data regarding women older than 60, there are few studies focusing on this age group concerning the prevalence of HPV, and no studies on the incidence of HPV and its association with dysplasia in this age group. The primary aim of the present study was to investigate the incidence of HPV and dysplasia in elderly women who had had an HPV negative test at the age of 60 years or older.

## Materials and methods

**Fig 1:**
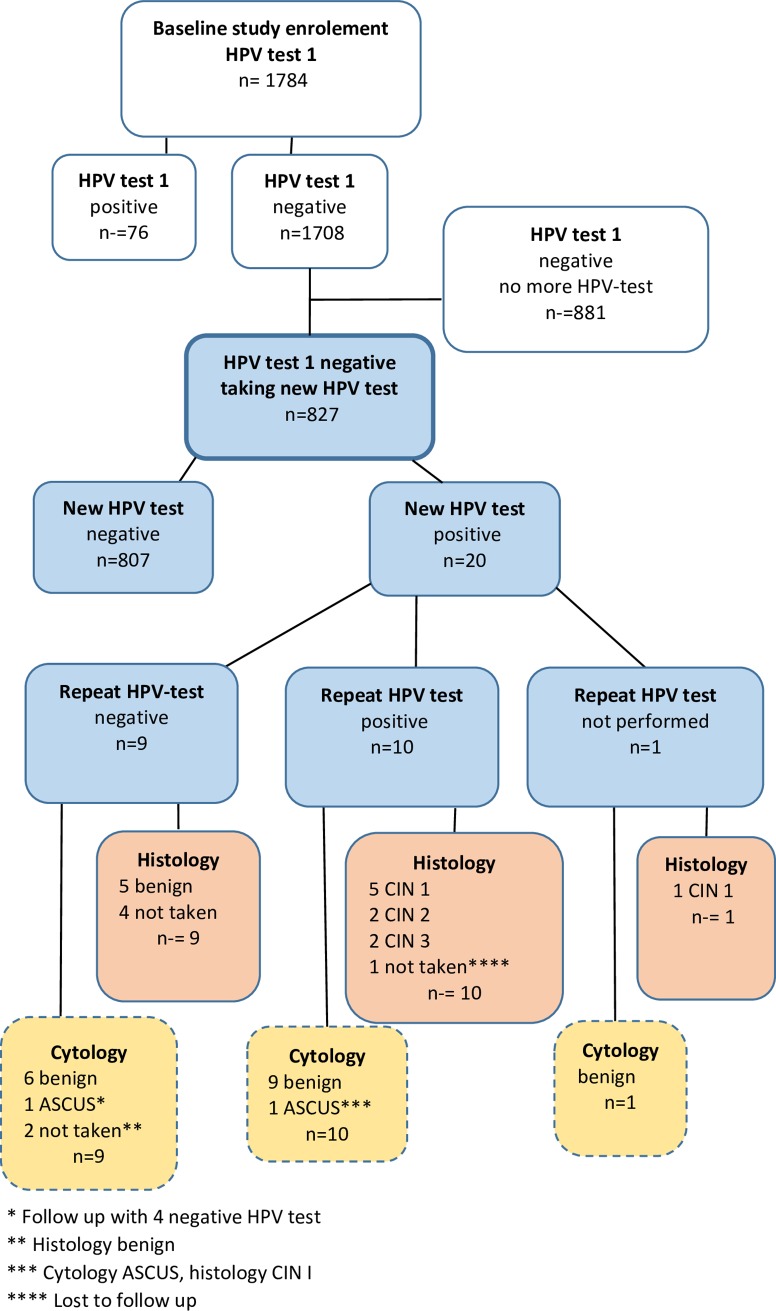
Flow chart showing study design and HPV and dysplasia occurrence.

This retrospective longitudinal descriptive study was based on 1784 women aged 60–90 years (mean age 68.2 years), attending an outpatient gynecology clinic in Dalarna County, Sweden, between 2004 and 2019, and having an HPV test as part of a gynecological examination (Fig 1). The study period was from September 2004 to May 2019. Women lacking a cervix were not included in the study. All women having a positive first HPV test were re-examined after 2.5 months on average, including a repeat HPV test, and cytology. All women who tested positive in the repeat HPV test were examined by colposcopy and sampling for histology with biopsies and/or cervical curettage and of the eleven women with dysplasia in histology ten women had a conization and one was scheduled for follow up with colposcopy. Women who tested negative in the repeat test were scheduled for a clinical follow up with a new HPV test after about a year. The primary endpoint measurements were HPV infection in women with a negative HPV test at the age of 60 or older, HPV after repeat test and precursor lesions verified by cytology and histology.

HPV (human papillomavirus) ASCUS (atypical squamous cells of undetermined significance), CIN 1–3 (cervical intraepithelial neoplasia grade 1–3).

PAP-smear on glass was performed 2004–2012 and LBC from 2013. All LBC specimens were screened by cytotechnicians, and those considered abnormal were reviewed by a surgical pathologist. For LBC, the Thin Prep^®^ Pap Test was used. The cervical smear was collected with a plastic spatula and a cytobrush. LBC specimens were placed in PreserveCyt solution and processed in the Thin Prep 5000-processor (HologicCytyc Corporation, Boxborough, Mass.) [15].

From 2004 until 2017 the terminology for the classification of cytology was for squamous cell lesions categorized into atypical squamous cells of undetermined significance (ASCUS), atypical squamous cells high-grade squamous lesion cannot be excluded (ASC-H), and cervical intraepithelial neoplasia grade 1–3 (CIN 1–3) [16]. For histology, the CIN classification was used. From 2018 the Bethesda system for classification cervical cytopathology and histology, with the classification low-grade intraepithelial neoplasia (LSIL) and high-grade intraepithelial neoplasia (HSIL), was used [17]. One senior pathologist reviewed the histology diagnosed as LSIL/HSIL and these results are presented as CIN.

Most of the HPV tests were performed using a multiplex real-time PCR assay (hpViR), which detects the following high-risk HPV types 16,18,31,33,35,39,45,51,52,56,58 and 59 (18 and 45 are detected together, and 33,52 and 58 as one group) [18]. A cervical sample was collected using a cytobrush. The sample was applied to a filter paper matrix, an indicating FTA elute card (Whatman, Inc., Clifton, New Jersey, rt no WB120411) [19, 20]. The threshold for a positive HPV type was set to 10 copies per PCR. At the beginning of the study, a few HPV tests were Hybrid Capture [21] and during the last year, a few HPV tests were Cobas© by Roche [22].

Specialists in surgical pathology examined the cervical histological samples for diagnosis. All cytology and histology specimens were examined at the Department of Pathology and Cytology, Falun County Hospital, Falun, Sweden.

For statistical analysis, Excel and the Statistical Package for Social Sciences (SPSS) version 22 for Windows were used. Confidence intervals (CI) of proportions (Fleiss) were calculated using Excel [23]. The study was approved by the Swedish Ethical Review Authority (Dnr: 2019–02489).

## Results

All 1784 women who were offered a test, accepted an HPV test. Only three HPV tests contained insufficient material for the HPV assay and those women had a new sample that was considered adequate and analyzed. The age at the first HPV test was 68.2 years in mean (60–89 years) in the whole group. The overall prevalence of HPV was 5.4%, (95%CI 4.4–6.6, 96/1784). Of the 1708 HPV-negative women, 827 had two or more HPV tests. The incidence of HPV in the 827 women, who were HPV negative in their first test, was 2.4% (95%CI 1.5–3.8, n = 20). The 20 women who became HPV positive were 67.8 years (range 60–74) at the first test that was HPV negative, and 71.5 years in mean (range 64–80) at the time of the first HPV positive test. The time from the HPV negative test to the first HPV positive test was, on average, 38 months (range 3–96).

At the repeat HPV test, on average 2.5 months later, 52.6% (10/19) were still HPV positive.

This means that 1.2% remained positive (95%CI 0.6–2.3, n = 10/827). Of the eight women who were HPV negative in the repeat test, six were HPV negative at the one year follow up, one had HPV 16 and benign cytology and histology, and one was lost to follow up. HPV 16 was the most common HPV type. One woman had multiple infection. As the colposcopic findings were inadequate and none of the women had a fully visible transformation zone, sampling for histology by cervical curettage and random biopsies was carried out. HPV-related dysplasia diagnosed by histology was found in 1.2% (95%CI 0.6–2.3, n = 10) of the 827 women. CIN2+ was found in 0.5% (95%CI 0.2–1.3, n = 4). Table 1. The positive predictive value (PPV) for any dysplasia was 50% (10/20) after the first HPV test and 100.0% (9/9) after the repeat HPV test. The PPV for CIN2+ was 20.0% after the first positive HPV test and 40.0% after the positive repeat HPV test. The period from an HPV negative test to HPV positive test and CIN2+ was on average 45.5 months (range 10–85 months). HPV 16 was found in 50% (2/4) of the high-grade lesions. All women with CIN2+ had normal cytology. No cancer or glandular dysplasia was detected.

**Table 1:** HPV, cytology, and histology in HPV-negative older women testing HPV- positive in follow up HPV test (n = 20).

ID	Age HPV neg	Age HPV positive	First positive HPV test	Repeat HPV test	Cytology	Histology
1	60	66	16	negative	benign	benign
2	70	73	16	16	benign	CIN 3
3	74	75	16	positive***	benign	CIN 1
4	67	74	16	16	benign	CIN 2
5	66	69	16	negative	Ascus**	*
6	76	80	16	16	benign	CIN 1
7	76	80	16	16	benign	CIN 1
8	61	64	16	negative	benign	benign
9	62	64	16, 33/52/58	negative	benign	*
10	70	78	31	31	benign	CIN 1
11	70	72	31	negative	benign	*
12	68	71	35	35	benign	*
13	70	73	51	negative	*	benign
14	69	72	56	negative	benign	benign
15	67	69	56	negative	benign	*
16	61	67	59	positive***	benign	CIN 3
17	60	64	59	*	benign	CIN 1
18	72	74	18/45	positive***	Ascus	CIN 1
19	66	67	33/52/58	33/52/58	benign	CIN 2
20	71	77	positive***	negative	*	benign
Age, mean	67.8	71.5				
Age, range	60–74	64–80				

* not performed

** the woman had follow-up with 4 HPV negative tests

***Cobas©test

## Discussion

High screening participation in the population is essential for optimal prevention of cervical cancer. Including older women may reduce the incidence and mortality in cervical cancer. There is no consensus on what age to stop screening for cervical cancer [24]. “WHO is working to ensure that all girls globally are vaccinated against HPV and that every woman over 30 is screened and treated for pre-cancerous lesions. Now is the time for global elimination” (https://www.who.int/docs/default-source/cervical-cancer/cervical-cancer-elimination-strategy.pdf?sfvrsn=8a083c4e_0). The expert opinion in the European Guidelines stated that screening for cervical cancer can be stopped at 60–64 years, given that the woman has had a recent negative test [14]. The level of evidence for this recommendation is low due to the lack of studies focusing on the prevalence and incidence of HPV infection in the elderly. Also, the consequence of the latency of HPV infection in the elderly is not known.

To our knowledge, this is the first longitudinal study on HPV in elderly women. We found that the incidence of HPV prevalence was low in older women who tested HPV negative at the age of 60 or older, but if they tested positive in a repeat HPV test, the risk of cervical dysplasia was very high. Since dysplasia was not detected by cytology in the vast majority of cases, LBC does not seem to be an appropriate method for screening in women older than 60. In our part of Sweden, women were screened with cytology up to the age of 60 until 2018, when primary HPV testing up to 64 years of age was introduced. The current study focused on women who were no longer screened. The strength of this study is the follow up with HPV testing, the same method of sampling, a validated HPV DNA test, repeat testing and diagnosis with colposcopy, cytology, and histology of all cases and over time.

The routine for follow up of HPV-related CIN 1 or HPV persistence with or without dysplasia is with a new HPV test and cytology or colposcopy with biopsies. In a recent study from Uppsala, diagnostic conization showed a high frequency of dysplasia in HPV-positive women, where 15% (6/40) of women older than 40 years of age, were revealed to have an undiagnosed CIN2+ when LEEP was performed [25].

A limitation of the study is that the biopsies of the portio were random as the transformation zone was not visible on the cervix, but in almost all cases a curettage of the cervix was performed. Whether the HPV test is a consequence of latent infection or a newly acquired infection is not known. The lead-time for development of dysplasia and cancer in a latent, respectively a newly acquired, infection in older women is not known.

It has been hypothesized that reactivation of HPV may be at least partly responsible for the high HPV prevalence observed in older women [26] [27]. Another possibility worth considering is changes in sexual behavior of men and/or women in middle-age [28, 29]. Korostil et al estimate that about 1 in 10 women and men in Australia who appear to have cleared HPV-16 infection may be latently infected [30].

## Conclusions

The incidence of HPV in elderly women who had an HPV negative test at the age of 60 years or older is low, but the PPV for dysplasia diagnosed by histology is high in those who remain HPV positive at a repeat test, and the time for the development of high-grade precursor lesions was relatively short. It is not possible to draw conclusions if these infected women have new or latent infections. We hope that our results will serve as motivation to conduct studies focusing on the appropriate age to stop screening older women in order to effectively reduce the prevalence of cervical cancer in this age group.

## Supporting information

S1 Dataset (XLSX)

S2 Dataset (XLSX)
